# Designing Better Exposure Notification Apps: The Role of Persuasive Design

**DOI:** 10.2196/28956

**Published:** 2021-11-16

**Authors:** Kiemute Oyibo, Plinio Pelegrini Morita

**Affiliations:** 1 School of Public Health Sciences Faculty of Health University of Waterloo Waterloo, ON Canada; 2 Department of Systems Design Engineering University of Waterloo Waterloo, ON Canada; 3 eHealth Innovation Techna Institute University Health Network Toronto, ON Canada; 4 Institute of Health Policy, Management, and Evaluation University of Toronto Toronto, ON Canada

**Keywords:** contact tracing app, exposure notification app, COVID Alert, COVID-19, persuasive technology, behavior change

## Abstract

**Background:**

Digital contact tracing apps have been deployed worldwide to limit the spread of COVID-19 during this pandemic and to facilitate the lifting of public health restrictions. However, due to privacy-, trust-, and design-related issues, the apps are yet to be widely adopted. This calls for an intervention to enable a critical mass of users to adopt them.

**Objective:**

The aim of this paper is to provide guidelines to design contact tracing apps as persuasive technologies to make them more appealing and effective.

**Methods:**

We identified the limitations of the current contact tracing apps on the market using the Government of Canada’s official exposure notification app (COVID Alert) as a case study. Particularly, we identified three interfaces in the COVID Alert app where the design can be improved. The interfaces include the no exposure status interface, exposure interface, and diagnosis report interface. We propose persuasive technology design guidelines to make them more motivational and effective in eliciting the desired behavior change.

**Results:**

Apart from trust and privacy concerns, we identified the minimalist and nonmotivational design of exposure notification apps as the key design-related factors that contribute to the current low uptake. We proposed persuasive strategies such as self-monitoring of daily contacts and exposure time to make the no exposure and exposure interfaces visually appealing and motivational. Moreover, we proposed social learning, praise, and reward to increase the diagnosis report interface’s effectiveness.

**Conclusions:**

We demonstrated that exposure notification apps can be designed as persuasive technologies by incorporating key persuasive features, which have the potential to improve uptake, use, COVID-19 diagnosis reporting, and compliance with social distancing guidelines.

## Introduction

The COVID-19 pandemic, beginning in the early part of 2020, has led to the development and deployment of several digital health technologies to slow the spread of COVID-19. COVID-19 is a human-to-human transmittable respiratory disease caused by the coronavirus known as SARS-CoV-2, which emerged in December 2019. Its symptoms include cough, sore throat, and high fever, which have the potential to cause pneumonia and respiratory failure [1]. Most prevalent among the technologies aimed at curbing COVID-19 are digital contact tracing apps, which help public health authorities to track or notify individuals who may have come into close contact with a person who is infected. Traditionally, contact tracing has been a manual process whereby people, potentially exposed to a human-to-human transmittable disease, are identified by interviewing persons who are infected with whom the former may have had close contact [2]. However, with the advancement in mobile technology and privacy-preserving cryptography (eg, the Google/Apple Exposure Notification system), the practice of contact tracing has gone predominantly digital worldwide [3]. Digital contact tracing does not replace manual tracing techniques but augments it to fast-track the containment of COVID-19 [4,5]. The main advantage of digital over manual contact tracing is that it automates the labor-intensive process, especially in situations where there are a limited number of human contact tracers [2,6]. Digital contact tracing, if adopted by a critical mass of people, is more likely to be faster, more effective, and accurate in comparison to the fallible nature of human memories, especially given that COVID-19 infection may be asymptomatic for up to 14 days [7].

**Figure 1:**
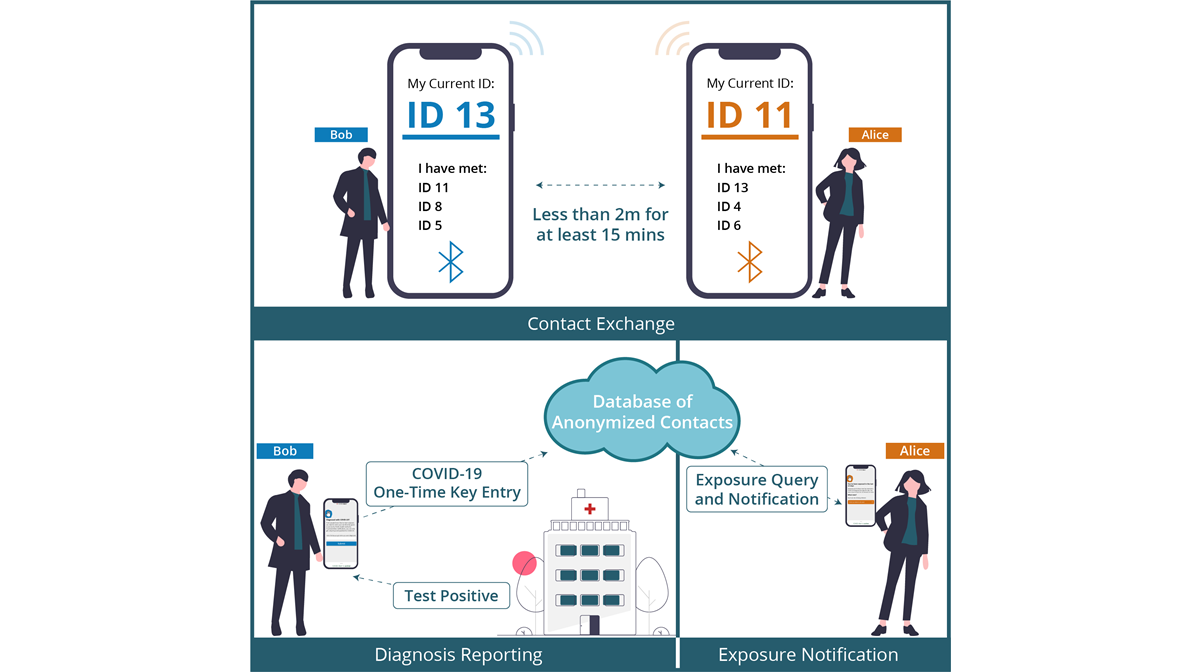
COVID-19 contact tracing and exposure notification process (adapted from Fairbank et al [8]).

Figure 1 shows how the exposure notification app works in the real world. If Bob and Alice come in close contact (ie, within a 2-meter distance) for 15 minutes or more, both contacts exchange a dynamic randomly generated identification number. In the future, if Bob tests positive and uploads his one-time key given to him by the public health authority to the cloud-based database of anonymized contacts, Alice will be contacted via the app and advised on what to do next.

Several countries worldwide, such as Australia, Canada, France, South Africa, and Singapore [9-11], have launched nationwide exposure notification apps in their respective official languages. The apps alert people who may have come in close contact with persons infected with COVID-19 for 15 minutes or more in the last 14 days. The Government of Canada’s exposure notification app is called “COVID Alert” [12]. It is available in two languages (English and French) and can be downloaded from the Apple and Android stores by Canadian residents in the Northwest Territories, Prince Edward Island, Nova Scotia, Quebec, Manitoba, Saskatchewan, New Brunswick, Ontario, and Newfoundland and Labrador [13]. Given the current poor uptake of contact tracing apps in general [14], in this paper, we used the COVID Alert app as a case study to uncover some of the weaknesses in the current design of most exposure notification apps on the market and demonstrate how persuasive features can be incorporated in their design to improve their persuasiveness, uptake, and effectiveness.

The rest of the paper is organized as follows. We begin by covering the poor uptake and design of contact tracing apps on the market and the need to make them more motivationally appealing. We then focus on persuasive design, key persuasive strategies relevant to contact tracing apps, and incorporating persuasive design in exposure notification apps using the COVID Alert app as a case study. Finally, we discuss the potential benefits of the proposed persuasive design of exposure notification apps and the ethics of persuasive technology.

## Poor Uptake of Current Exposure Notification Apps

The Canadian Government has widely publicized the COVID Alert app, but acquiring a critical mass of users has been hampered due to privacy concerns, trust, and human factor design issues. Part of the adoption campaign involved Prime Minister Justin Trudeau urging Canadian residents, especially young people, to download and use the COVID Alert app to improve contact tracing and diminish disease trajectories [13]. In 2020, it was estimated that there were 31.38 million smartphone users in Canada [15]. Yet, as of November 26, 2020, the COVID alert app has only been downloaded about 5.5 million times from both Apple and Google stores [16]. This means (assuming each download can be associated with a unique smartphone user) approximately 17.5 percent of the smartphone users in Canada in 2020 downloaded the app as of November 26. The low adoption rate of the COVID Alert app among the Canadian population limits its effectiveness, as research shows that 56% of the population would have to use the app to considerably slow down the spread of the virus [17].

## Problems With Current Contact Tracing and Exposure Notification Apps

There are several problems associated with the low uptake of contact tracing and exposure notification apps worldwide. Concerns hindering their adoption are privacy, data use, public surveillance, poor persuasive design, and lack of customization to mention but a few [7,18].

**Figure 2:**
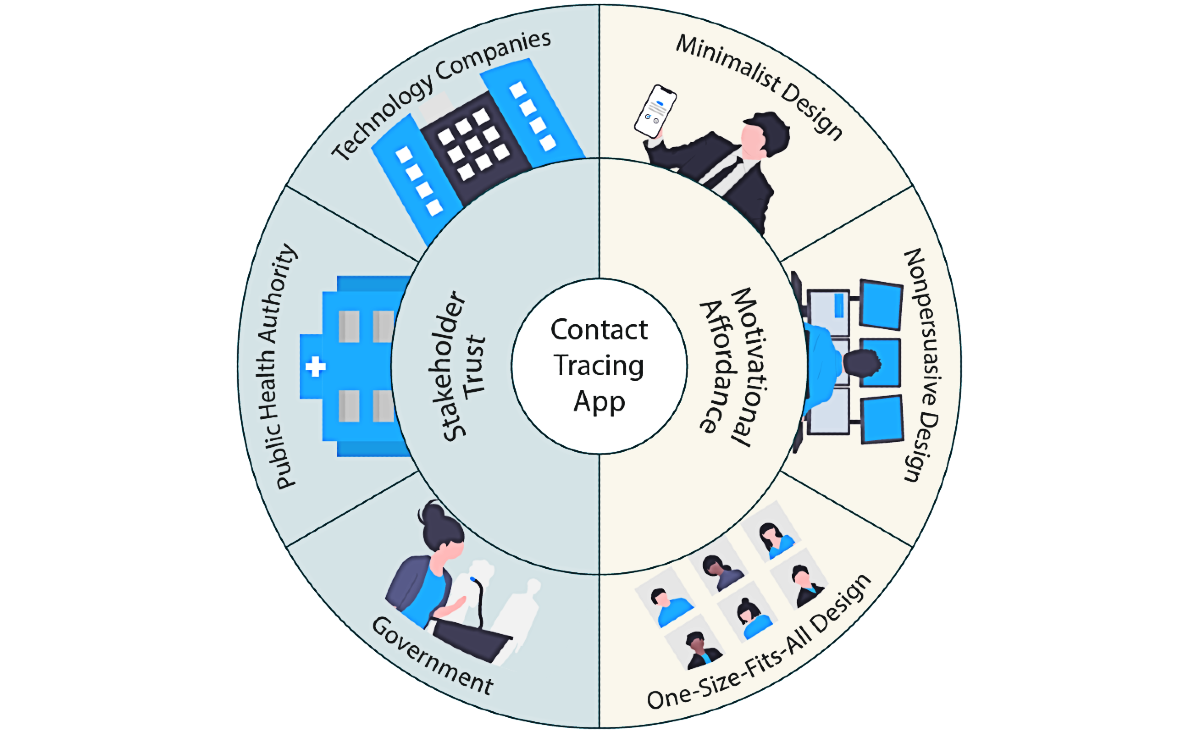
Stakeholder and design-related issues surrounding the low uptake of contact tracing and exposure notification apps.

Broadly, these problems can be grouped into two categories, as shown in Figure 2. The first category is lack of trust in stakeholders (eg, government, tech companies, or public health authority) pertaining to data privacy and protection [19-21]. The second category is the lack of motivational affordances in the user interface (UI) design of exposure notification apps. In other words, these apps are minimalist, nonpersuasive, and use a one-size-fits-all approach, which can negatively impact adoption [20,22].

### Lack of Trust in Contact Tracing Stakeholders

Privacy and trust-related concerns have been raised by the public concerning how COVID-19 health and tech stakeholders will handle users’ privacy and data [7]. For example, most Americans may trust COVID-19 stakeholders such as public health agencies and universities, but they do not trust tech companies such as Apple and Google, which developed the privacy-preserving Google/Apple Exposure Notification system, which most of the contact tracing apps on the market require and support to function properly [12]. A cross-section of US smartphone users was asked the question, “How much, if at all, do you trust _____ to ensure that people who report being diagnosed with coronavirus using their smartphone app remain anonymous — a great deal, a good amount, not too much or not at all?” A total of 56% of those polled (ie, nearly 3 in 5) did not trust tech companies such as Apple and Google, but 57% and 56% trusted public health agencies and universities a great deal or a good amount, respectively [23]. The limited trust in tech companies such as Apple and Google (<45%) may not come as a surprise given the widely reported Facebook-Cambridge Analytica Scandal about the 2016 United States elections [24].

### Lack of Motivational Affordances in Exposure Notification Apps

High uptake is crucial for exposure notification apps to be effective in mitigating the spread of COVID-19. However, according to Walrave et al [25], “*it* remains unclear how we can motivate citizens to use these apps.” Although the government and tech companies have taken some measures to increase public trust by way of decentralization of collected data [12], Bluetooth contact tracing, and nontracking/storage of users’ location data via global positioning technology, much is yet to be done in the area of persuasive design to increase the adoption rate. For example, the current version of the COVID Alert app is minimalist [26] and lacks motivational affordances and incentives [27]. Motivational affordances are the persuasive elements that satisfy users’ needs. According to Zhang [28], when an information and communication technology (ICT) satisfies users’ motivational needs, they feel enjoyment and want it more. Hence, “the ultimate goal of designing an ICT for human use is to achieve high motivational affordance so that users would be attracted to it, really want to use it, and cannot live without it” [28]. However, “[a]part from providing receiving notifications about possible infections, current contract tracing apps appear to not provide a clear benefit to the user” [29]. Specifically, most of them lack vital persuasive features that motivate people to use digital health technologies to monitor and manage their health behaviors. Hence, the lack of persuasive features may contribute to low adoption rates of many contact tracing and exposure notification apps on the market [30].

Digital health researchers have stated that incorporating persuasive features into contact tracing apps could increase their adoption and use by the wider population [27]. In other words, contact tracing apps are more likely to be effective as persuasive technologies than as traditional information systems focused on functionality.

**Figure 3:**
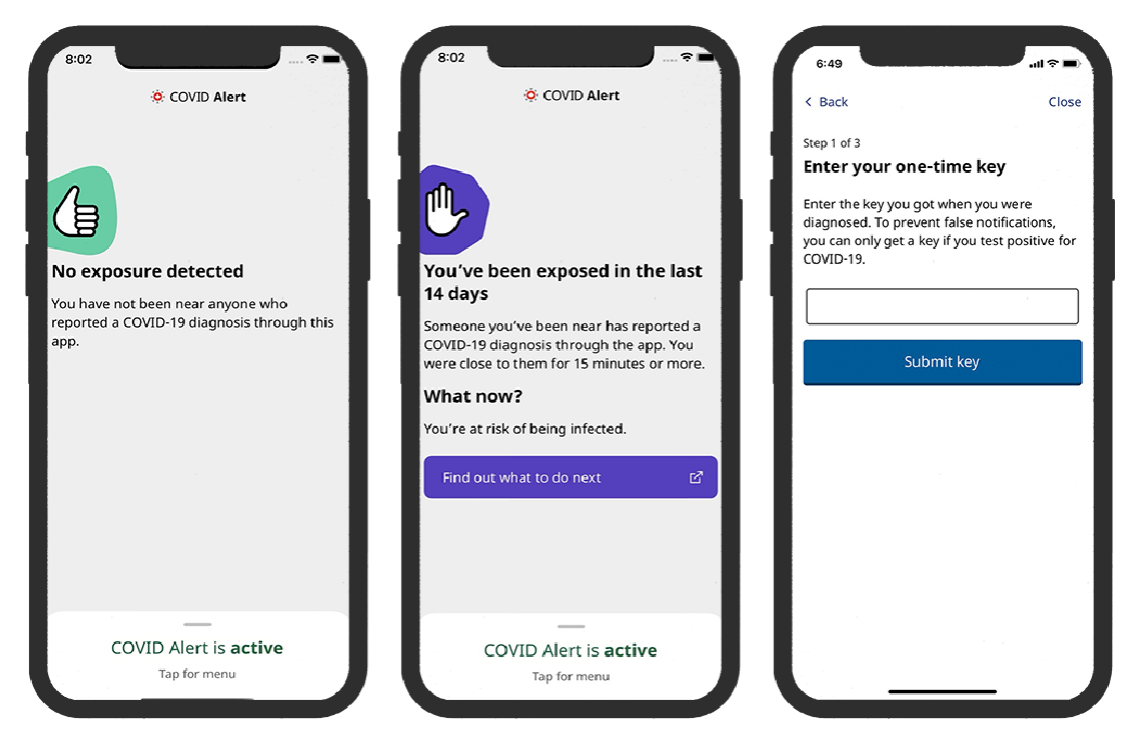
Key user interfaces in the COVID Alert app (Government of Ontario [32]).

Persuasive technology is an interactive system intentionally designed to change attitudes or behaviors positively through persuasion and social influence but not through coercion or deception [31]. However, the current version of the COVID Alert app lacks basic persuasive and social influence principles that can motivate more users to download and use the app more frequently. Figure 3 shows the three main functional UIs of the COVID Alert app: “No Exposure,” “Exposure,” and “Diagnosis Report.” Apart from being minimalistic, all three UIs do not support essential persuasive features such as monitoring of the users’ daily contacts and exposure time. This may help them regulate themselves concerning observing social (physical) distancing guidelines in public settings.

## Persuasive Design

Persuasive design involves applied social psychology theories in the design of technologies to change behaviors and attitudes. Hence, persuasive technology, also called “Captology” by Fogg [31], is regarded as the intersection of computer systems (from the field of human-computer interaction) and the art of persuasion (from the field of psychology). A typical example of a persuasive technology is a mobile fitness app aimed at motivating people to exercise more to improve their mental well-being and physical fitness. Persuasive design focuses on influencing human behavior, attitude, motivation, and compliance through the systematic design of a system’s features and affordances to promote behavior change.

### Persuasive Techniques

**Figure 4:**
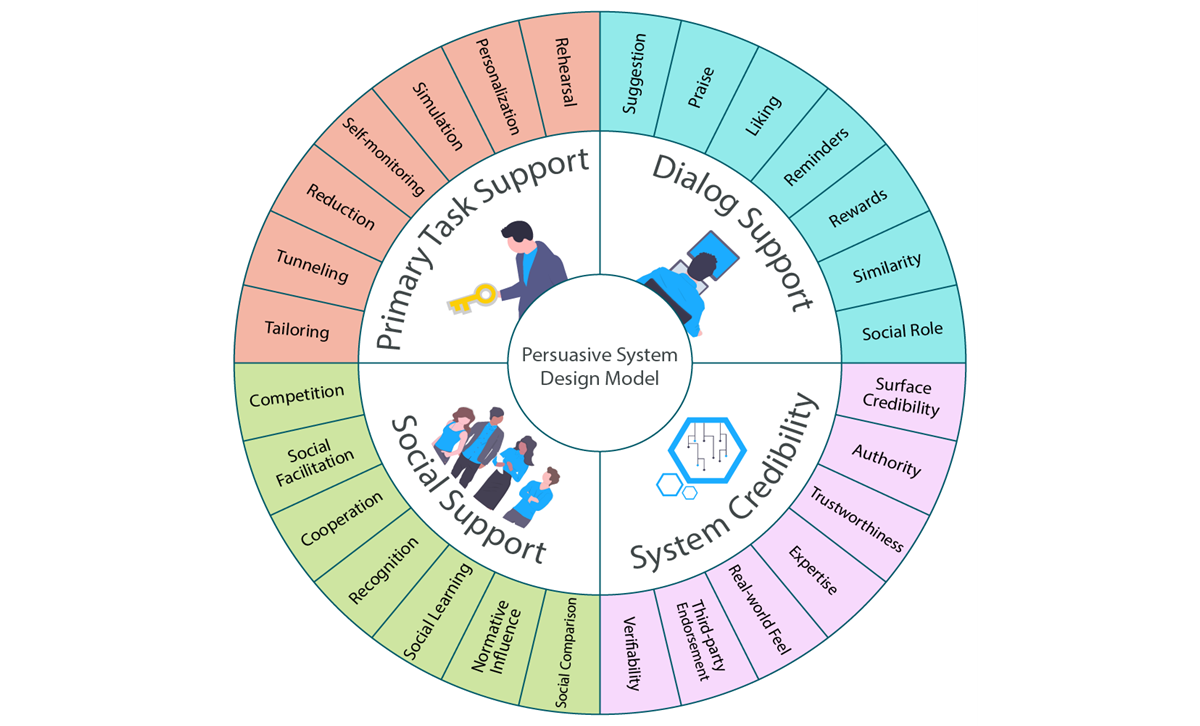
Persuasive system design model [36,37].

There are two main design frameworks commonly used in designing and evaluating persuasive technologies. The first framework is called Cialdini’s [33] principles of persuasion, which comprise six persuasive techniques: authority, commitment, reciprocity, liking, consensus, and scarcity [34,35]. The second framework is called the persuasive system design model [36], which comprises 28 persuasive techniques and extends Fogg’s [31] seven persuasive techniques. The persuasive system design model includes four broad categories (primary task support, dialogue support, system credibility support, and social support) as shown in Figure 4 [36,37].

First, primary task support includes persuasive techniques that help the user to carry out the target behavior easily and effectively. Second, dialogue support includes persuasive techniques that motivate the user to perform the target behavior through feedback and interaction with the persuasive application. Third, social support includes persuasive techniques that motivate the user to carry out the target behavior through social influence. Finally, system credibility support includes persuasive techniques that make the persuasive application look credible to the user [38].

**Figure 5:**
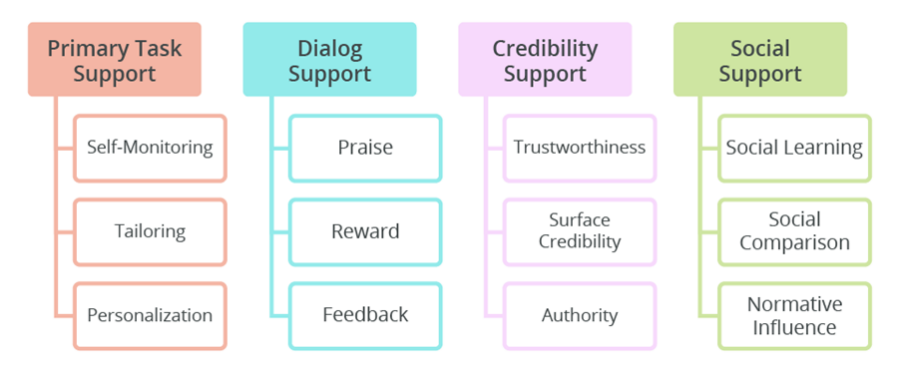
Twelve contact tracing app persuasive techniques from the persuasive system design model.

Each of the four categories in the persuasive system design model comprises seven persuasive techniques. Figure 5 shows three persuasive techniques in each of the four categories relevant to contact tracing apps. For example, primary task support comprises self-monitoring, tailoring, and personalization, and social support includes social learning, social comparison, and normative influence. These techniques, widely studied in persuasive technology research, have proven effective in changing health behaviors such as physical activity [39,40]. Moreover, dialogue support comprises praise, reward, and feedback. In particular, reward, be it virtual, tangible, or monetary, holds potential in motivating behavior change, as people from both high-income and low-income countries are receptive to it [41]. Finally, credibility support comprises trustworthiness, surface credibility, and authority. Research [36] shows that persuasive apps perceived as trustworthy and credible are more likely to motivate behavior change. Prior studies found a direct or indirect relationship between source trustworthiness [42] or perceived credibility [43] and behavioral intentions. Moreover, Oyibo et al [44] found that people from both high-income and low-income countries are receptive to the authority strategy. Interestingly, current exposure notification apps on the market are already equipped with the authority and credibility strategies by default given that they were sponsored by national governments that symbolize authority. However, the issue of trust in the area of data protection and privacy remains a roadblock to adoption [23].

### Example Implementation of Key Persuasive Design Techniques

**Figure 6:**
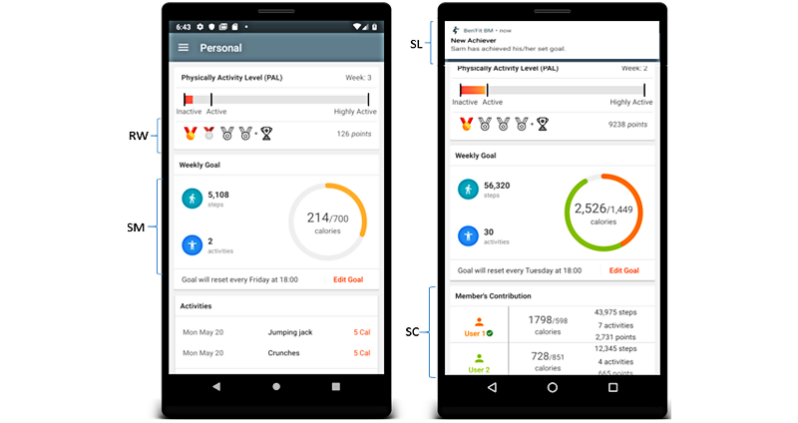
Implementation of SM, RW, SC, and SL in a fitness app aimed at promoting physical activity [46]. RW: reward; SC: social comparison; SL: social learning; SM: self-monitoring.

**Figure 7:**
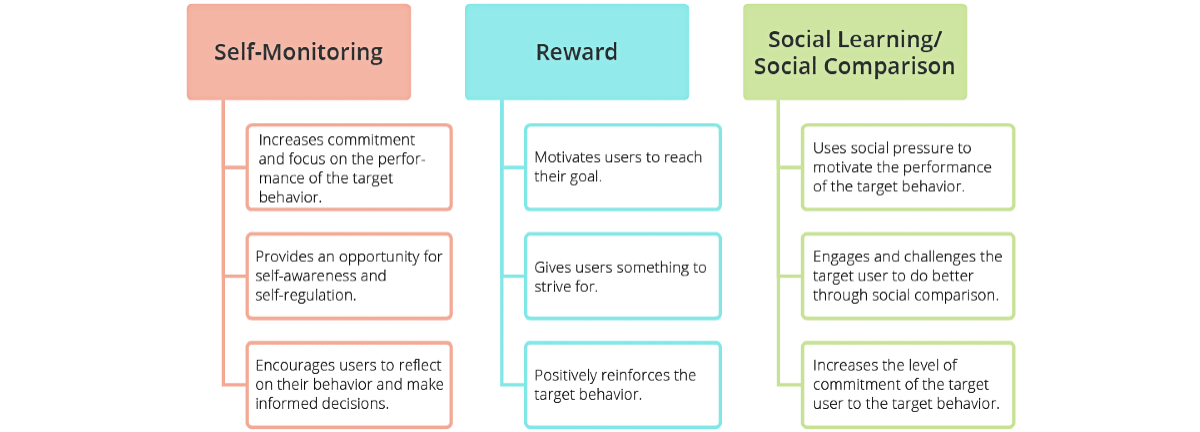
Advantages of self-monitoring, reward, social learning, and social comparison [47].

Persuasive techniques are implemented in most mobile health apps on the market to motivate behavior change and help users achieve their goals. Figure 6 shows a fitness app called “BEN’FIT,” in which reward/self-monitoring and social learning/social comparison are, respectively, implemented in the personal and social versions (Oyibo et al [45]). Self-monitoring enables the user to track their physical activity, including calories burned and step count over time. Regarded as the cornerstone of persuasive apps, self-monitoring fosters self-awareness and commitment, among other advantages shown in Figure 7 [46]. In the context of contact tracing apps, Cruz et al [47] found that over 50% of their surveyed participants wanted to know how many infected people they have come in contact with and how many infected people have passed through a given location. Reward provides users with something to strive for and reinforces behaviors [48]. Feedback allows the user to get important information about their behavior at specific points in time, for example, after achieving a 10,000 steps milestone. Feedback is not listed as a dialogue support feature in the persuasive system design model, yet it is used as a persuasive feature in motivating behavioral change. Social learning and social comparison, which are correlated [49], use social pressure to motivate the target behavior [48].

## Incorporating Persuasive Design in Exposure Notification Apps

**Figure 8:**
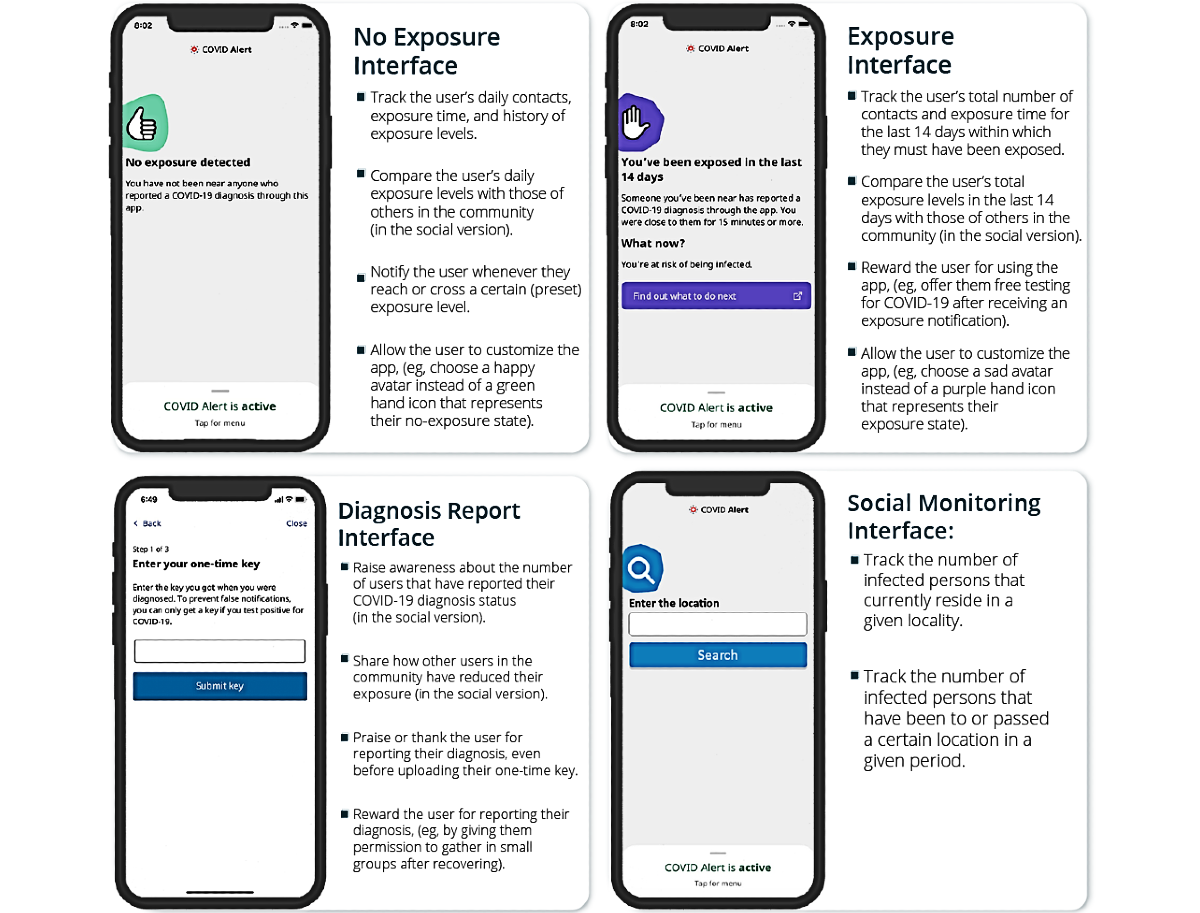
Guidelines for incorporating persuasive features into key user interfaces of exposure notification apps using COVID Alert as a case study.

The COVID Alert app can be redesigned to be more appealing and motivating to the target users by incorporating essential persuasive features to increase its effectiveness. Figure 8 provides guidelines for integrating persuasive features such as self-monitoring, praise, reward, social comparison, and social learning. However, prior research in the physical activity domain shows that Canadians are more likely to be receptive to personal than social strategies [50]. For this reason, there should be a personal and a social version of the app to enable the target users to make a choice based on their preferences.

### No Exposure Interface

In the no exposure UI, a self-monitoring feature, which tracks daily contacts and exposure time, and showcases historical behavior, can be incorporated in the second half of the screen, which is currently blank. The implementation of the self-monitoring feature is presented in Oyibo et al [51]. In the social version, a social comparison feature, which compares the user’s exposure levels (daily contacts and exposure time) with those of others in the community, can be incorporated as well. In addition, users can be allowed to customize the app (eg, choose a happy face avatar instead of a green hand icon that represents their no exposure state). Research shows that well-designed avatars can improve the user experience by drawing a closer connection between the user’s lived and digital identities as, for example, avatars possess some human signifiers like facial expressions that convey emotion [52]. This is in line with the liking principle in the persuasive system design model (Figure 4), which states that people are more likely to be persuaded by people similar to them or that are attractive [33,36].

### Exposure Interface

In the exposure UI, a self-monitoring feature, which tracks the total number of contacts and approximately when the user was exposed, can be incorporated in the middle of the screen, as shown in Figure 8. The implementation of the self-monitoring feature is presented in Oyibo et al [51]. As in the no exposure UI, users should be able to customize the app (eg, choose a sad face avatar instead of a purple hand icon to represent their exposed state). In addition, in the social version, they should be given the choice to compare their exposure levels with those of others in the community as an additional means of motivation and insight.

### Diagnosis Report Interface

In the diagnosis report UI, a social learning feature, which informs the user about the number of persons that have reported their COVID-19 diagnosis for a given period (eg, day or week), can be incorporated in the middle of the screen as shown in Figure 8. This additional statistical information can encourage users, when infected, to report their diagnosis to ensure the safety of the community. The implementation of the social learning feature is presented in Oyibo et al [51]. Moreover, users can be praised or rewarded for reporting their diagnosis. In a recent study, Jonker et al [53] found that respondents preferred apps that offer them incentives such as a token monetary reward (€5 [US $6] or €10 [US $12] a month), permission to gather in small groups (eg, after recovering), or free testing for COVID-19 after receiving an exposure alert.

### Social Location Monitoring Interface

In addition to the 12 persuasive features drawn from the persuasive system design model (Figure 4), hot spot monitoring, which we call “Social Location Monitoring,” can be used as a persuasive strategy to promote adoption and use. Social location monitoring is the tracking and gathering of information about a location that includes the number of persons who are infected that currently reside in, have been to, or passed the location in a given period to help users make informed decisions. Figure 8 shows a hypothetical interface for incorporating social location monitoring to motivate beneficial behaviors (eg, avoiding hot spots, social distancing, and wearing a mask). In a recent study, Li et al [54] found that respondents were more willing to install contact tracing apps that collect users’ location data than those that do not, due to the additional benefits they provide about hot spot information and analysis. Social location monitoring can help local authorities allocate resources in a better way and enact better health care policies during the COVID-19 pandemic [55].

## Potential Impact of the Proposed Persuasive Design

The projected impact of the persuasive design of exposure notification apps includes improved uptake, frequent use, increased report of diagnosis, and compliance with social distancing guidelines. In future research efforts, we hope to implement these persuasive design guidelines and conduct a study to investigate the effectiveness of the persuasive design of exposure notification apps using the COVID Alert app as a case study. Although research has shown that persuasive design can promote behavior change (eg, in the physical domain or health eating), it is still not certain whether the proposed persuasive design guidelines for exposure notification apps can promote the target behaviors. Hence, there is a need for empirical research in the future to investigate the effectiveness of the proposed persuasive system design guidelines.

## Ethics of Persuasive Design

Ethical concerns about the app and impact of persuasive design have been raised in the gray and academic literature. Admittedly, in the wrong hands, persuasive design can be exploited or used to manipulate unsuspecting users for financial and other gains [56]. We regard this as “persuasive design for unethical gains.” One area that experts believe that persuasive technologies have been unethically used is digital apps for children. Research shows that the amount of kids’ screen time in 2018 was 10 times the amount in 2011, with kids spending an average of 6 hours and 40 minutes using persuasive technologies such as game apps and social media. Hence, some health professionals believed “children’s behaviors are being exploited in the name of the tech world’s profit” [56]. This led 50 psychologists in 2018 to send a letter to the American Psychological Association (APA) “accusing psychologists working at tech companies of using ‘hidden manipulation techniques’ [and prevailing on] the APA to take an ethical stand on behalf of kids” [56]. However, leveraging persuasive design for financial gains or unethical benefits is not what “persuasive design for behavior change” is about. Rather, the sole purpose for persuasive design for behavior change is to support the user in adopting and performing behaviors beneficial to themselves or society. An example of behavior change beneficial to the individual is eating healthy or exercising regularly. A persuasive app can be used to promote these behaviors. An example of such an app is “List It” [57]. The app motivates users to select healthy options from a shopping list. Moreover, a behavior change beneficial to the society is commuting by public transportation (eg, bus or train) instead of driving one’s personal car [58]. Broadly speaking, eco-friendly behaviors aimed at reducing carbon footprints will help, on a large scale, reduce global warming and climate change [59]. An example of a persuasive app aimed at reducing carbon footprints is “EcoIsland” [60]. The app, which supports the feedback strategy, encourages users to perform eco-friendly activities (turning down the room heater by 1 °C, commuting by train instead of driving a car, etc) to reduce carbon dioxide emission. Overall, the guiding moral principle (also known as the golden rule) of persuasive technology is that “designers of persuasive technology should not create any artifact that persuades someone to do or think something that they (the designers) would not want to be persuaded of themselves” [61].

## Conclusions

In this paper, we identified some of the issues surrounding the low uptake of contact tracing and exposure notification apps deployed by national governments worldwide to curb the spread of COVID-19 and speed up the lifting of public health restrictions. Specifically, we pinpointed lack of trust, concerns about privacy and data use by COVID-19 stakeholders, and the nonmotivational design of contact tracing and exposure notification apps as potential reasons for the low adoption rates worldwide. Using the Government of Canada’s COVID Alert app as a case study, we provided persuasive technology design guidelines that can help incorporate persuasive features in contact tracing and exposure notification apps to increase their uptake, frequent use, and compliance with social distancing guidelines. For example, we identified three use cases (no exposure status, exposure status, and diagnosis report interfaces) that can support persuasive features such as self-monitoring of the number of daily contacts and COVID-19 exposure time, and social learning about other users that have reported their diagnosis over a given period. In future work, we hope to conduct a user study to investigate the effectiveness of the implemented guidelines among Canadian residents using the COVID Alert app as a case study [51].

## Abbreviations

APA: American Psychological Association
ICT: information and communication technology
UI: user interface
